# Surgical Instrument Segmentation via Segment-Then-Classify Framework with Instance-Level Spatiotemporal Consistency Modeling

**DOI:** 10.3390/jimaging11100364

**Published:** 2025-10-15

**Authors:** Tiyao Zhang, Xue Yuan, Hongze Xu

**Affiliations:** School of Automation and Intelligence, Beijing Jiaotong University, Beijing 100044, China; 22115028@bjtu.edu.cn (T.Z.); hzxu@bjtu.edu.cn (H.X.)

**Keywords:** surgical instrument segmentation, segment-then-classify framework, instance-level spatiotemporal consistency modeling

## Abstract

Accurate segmentation of surgical instruments in endoscopic videos is crucial for robot-assisted surgery and intraoperative analysis. This paper presents a Segment-then-Classify framework that decouples mask generation from semantic classification to enhance spatial completeness and temporal stability. First, a Mask2Former-based segmentation backbone generates class-agnostic instance masks and region features. Then, a bounding box-guided instance-level spatiotemporal modeling module fuses geometric priors and temporal consistency through a lightweight transformer encoder. This design improves interpretability and robustness under occlusion and motion blur. Experiments on the EndoVis 2017 and 2018 datasets demonstrate that our framework achieves mIoU improvements of 3.06%, 2.99%, and 1.67% and mcIoU gains of 2.36%, 2.85%, and 6.06%, respectively, over previously state-of-the-art methods, while maintaining computational efficiency.

## 1. Introduction

Minimally invasive surgery (MIS) has become a valuable alternative to conventional open procedures, such as appendectomy [1], cholecystectomy [2], and pancreas or liver resection [3], owing to its smaller incisions, reduced trauma, and faster recovery. However, MIS also poses unique challenges to surgeons, including a restricted field of view and complex hand–eye coordination, which collectively increase cognitive workload and demand high operational precision. To alleviate these challenges, accurate and temporally consistent surgical instrument segmentation is essential. Reliable segmentation facilitates real-time surgical navigation and robotic control, while also supporting postoperative analysis, skill assessment, and workflow optimization. Enhancing the robustness and interpretability of segmentation therefore directly contributes to improving the safety and automation level of computer-assisted MIS. Nevertheless, the surgical environment is highly dynamic—characterized by specular reflections, occlusions, motion blur, and cluttered anatomical structures—which makes robust and temporally stable segmentation particularly difficult to achieve. Traditional pixel-wise classification approaches often fail under these conditions, producing fragmented or temporally inconsistent masks that limit their usefulness in downstream tasks requiring temporal coherence and spatial completeness.

To address these challenges, Robot-Assisted Minimally Invasive Surgery (RAMIS) has been developed with great vigor in recent years [4,5], aiming to assist surgeons in overcoming these shortcomings more effectively, including the utilization of automatic surgical skill analysis [6], surgical stage segmentation [7], surgical scene reconstruction [8], field of view expansion [9,10], and other techniques. To accomplish these sophisticated operations, it is essential to accurately identify the location of surgical instruments within the image domain through the utilization of image segmentation methods.

In the early stages of development, techniques employed utilized handcrafted features derived from color and texture, in conjunction with machine learning models such as random forests and Gaussian mixtures [11,12]. Subsequently, convolutional neural network methods facilitated further advancement in the field of surgical instrument segmentation. ToolNet [13] employed a fully nested fully convolutional network to impose multi-scale prediction constraints. In a related vein, Laina et al. [14] put forth a multi-task convolutional neural network for parallel regression segmentation and localization. Milletari et al. [15] employed a residual convolutional neural network to integrate multi-scale features extracted from a frame through a long short-term memory (LSTM) unit.

Although deep learning–based approaches have achieved unprecedented success, most existing work treats surgical video data as independent static frames, relying solely on visual cues for segmentation. However, surgical videos contain rich temporal dynamics that can provide critical clues for improving accuracy and stability. Effectively exploiting these temporal cues and explicitly injecting them into the network has therefore become a key challenge. Recent transformer-based methods, such as MATIS [16], leverage Multiscale Vision Transformers [17] to extract global temporal consistency information from video sequences for mask classification.

In this study, we propose a segment-then-classify framework for surgical instrument segmentation in endoscopic videos. The proposed framework decouples mask generation and category classification, producing spatially coherent masks while improving classification stability. Specifically, we first generate class-agnostic instance masks using a Mask2Former-based segmentation backbone. Then, to enhance classification accuracy and temporal consistency, we introduce a bounding box-guided instance-level spatiotemporal consistency modeling module, which combines region proposal features with normalized bounding box priors. A lightweight Transformer encoder is employed to capture the temporal evolution of each instrument instance, ensuring consistent classification across consecutive frames.

The main contributions of this paper can be summarized as follows:

(1) We propose a segment-then-classify framework that decouples segmentation and classification, improving the spatial completeness and temporal stability of surgical instrument segmentation.
(2) We introduce a bounding box-guided temporal modeling strategy that combines spatial priors with semantic region features to enhance instance-level classification consistency.
(3) We achieve state-of-the-art performance on the EndoVis 2017 and 2018 datasets, demonstrating the effectiveness and interpretability of the proposed framework.

The remainder of this paper is organized as follows. Section 2 reviews related work on surgical instrument segmentation and temporal modeling approaches. Section 3 presents the details of the proposed framework. Section 4 reports the experimental results and analysis. Section 5 and Section 6 discusses the findings and open research questions, followed by conclusions in Section 7.

## 2. Related Work

### 2.1. Surgical Instrument Segmentation

Surgical instrument segmentation has been extensively studied as a core component of computer-assisted minimally invasive surgery. Early works primarily focused on pixel-wise classification using convolutional neural networks (CNNs), such as TernausNet [18] and U-Net-based models [19], which achieved remarkable success in static image segmentation. However, these methods often produce fragmented or incomplete masks when applied to video sequences, due to the high variability of surgical scenes and rapid instrument motion.

To improve mask quality, several methods adopted instance segmentation strategies that treat surgical instruments as independent objects rather than homogeneous pixel classes. For example, ISINet [20] introduced instance-level learning for object differentiation, while TernausNet [18] leveraged transformer-based attention mechanisms to integrate multi-scale features. MATIS [16], a recent representative work, proposed a masked-attention transformer to perform segmentation followed by classification, offering improved spatial coherence. Nevertheless, these approaches still exhibit limitations in temporal consistency—the classification of the same instrument may vary across frames due to motion blur, occlusion, or tool overlap.

In addition to the above methods, several recent works have explored transformer-based and foundation-model-inspired architectures for surgical scene understanding. For instance, AGMF-Net [21] introduced multi-scale temporal attention for video object segmentation; and Samsurg [22] leveraged the Segment Anything model for zero-shot instrument detection. These approaches highlight the trend toward integrating strong priors and global attention, which motivates our instance-level temporal consistency design.

The above methods highlight the need to go beyond frame-level segmentation and integrate temporal dynamics in an interpretable and instance-aware manner. This motivates our work, which aims to enhance instance-level temporal consistency by combining explicit geometric priors with transformer-based temporal modeling.

### 2.2. Temporal Modeling and Segment-Then-Classify Frameworks

Temporal modeling has emerged as an effective approach to improve consistency and robustness in video-based segmentation tasks. Traditional methods typically adopt optical flow estimation or recurrent architectures (e.g., ConvLSTM) to capture temporal dependencies between frames. However, these techniques are often computationally expensive and sensitive to motion noise, making them less suitable for real-time surgical applications.

Recently, transformer-based architectures have demonstrated strong potential for learning long-range temporal relationships. For instance, MF-TAPNet [23] and TraSeTR [24] utilize multi-frame attention to propagate contextual information across frames. While these approaches successfully model temporal information at the feature level, they often lack instance-level interpretability, making it difficult to ensure consistent classification of each instrument across frames.

In contrast, the segment-then-classify paradigm offers a promising alternative by decoupling spatial mask prediction from semantic classification. Instead of performing pixel-wise classification directly, the model first generates class-agnostic instance masks and subsequently classifies them using instance-level features. This decoupling facilitates better spatial completeness and provides flexibility for integrating temporal and positional priors. Our method builds upon this principle and extends it by introducing bounding box-guided instance-level spatiotemporal consistency modeling, which explicitly captures the geometric and temporal evolution of each instrument instance using a lightweight transformer encoder. This design enhances interpretability, reduces ambiguity in instance tracking, and improves classification stability under complex motion.

## 3. Materials and Methods

### 3.1. Overview

The proposed method aims to achieve spatially complete and temporally consistent segmentation of surgical instruments by decoupling mask generation and classification.

**Figure 1:**
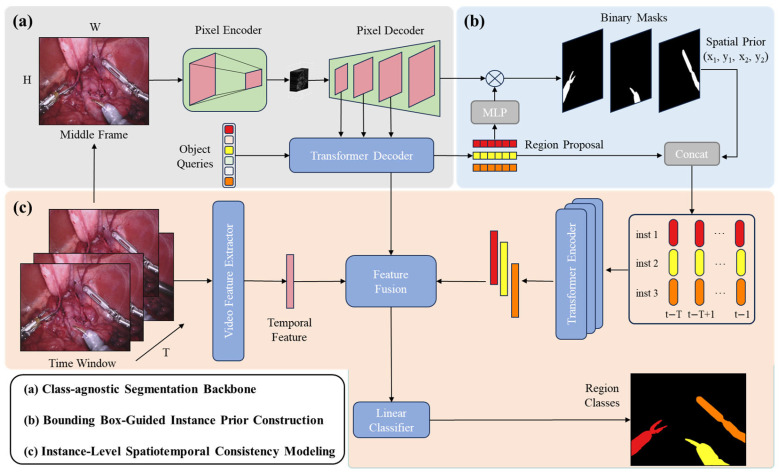
Overview of the proposed Segment-then-Classify framework for surgical instrument segmentation. The framework consists of three main stages: (**a**) a Mask2Former-based segmentation backbone to generate class-agnostic instance masks and region features; (**b**) a bounding box-guided instance prior construction module that fuses spatial priors with semantic features; and (**c**) an instance-level spatiotemporal consistency modeling module employing a transformer encoder to ensure consistent classification across consecutive frames.

As illustrated in Figure 1, the overall framework follows a segment-then-classify paradigm and consists of three main stages:

(1) a class-agnostic segmentation backbone based on Mask2Former to generate instance masks and corresponding region proposal features;
(2) a bounding box-guided instance prior construction module to combine spatial priors and semantic region features;
(3) an instance-level spatiotemporal consistency modeling module that employs a transformer encoder to capture temporal relationships among instances across consecutive frames.

The final classification head assigns semantic categories to each predicted mask.

### 3.2. Class-Agnostic Segmentation Backbone

We adopt Mask2Former as the segmentation backbone due to its ability to handle multiple instances with high spatial precision.

Given an input video frame $I_{t}\in R^{H\times W\times 3}$, Mask2Former extracts a feature map $F_{t}\in R^{C\times H\prime \times W\prime }$ through a pixel decoder and transformer-based mask decoder. A set of N instance queries is used to generate mask embeddings ${\left\{M_{t}^{i}\right\}}_{i=1}^{N}$ and corresponding region proposal features ${\left\{f_{t}^{i}\right\}}_{i=1}^{N}$.

The segmentation objective can be formulated as Equation (1):

(1) $$L_{seg}=\frac{1}{N}\sum_{i=1}^{N}\left[L_{dice}\left(m_{t}^{i},{\hat{m}}_{t}^{i}\right)+L_{bce}\left(m_{t}^{i},{\hat{m}}_{t}^{i}\right)\right],$$

where ${\hat{m}}_{t}^{i}$ is the predicted mask, and $L_{dice}$ and $L_{bce}$ denote the Dice loss and binary cross-entropy loss, respectively.

The output of this stage provides instance-level mask features $f_{t}^{i}$, which contain semantic and appearance information but lack explicit spatial priors or temporal awareness.

### 3.3. Bounding Box-Guided Instance Prior Construction

For each predicted mask ${\hat{m}}_{t}^{i}$, we compute its bounding box coordinates as Equation (2):

(2) $$b_{t}^{i}=\left(x_{min}^{i},\;y_{min}^{i},x_{max}^{i},y_{max}^{i}\right),$$

and normalize them by the frame dimensions, as shown in Equation (3):

(3) $${\overset{\sim }{b}}_{t}^{i}=\left(\frac{x_{min}^{i}}{W},\frac{y_{min}^{i}}{H},\frac{x_{max}^{i}}{W},\frac{y_{max}^{i}}{H}\right),$$

Each normalized bounding box encodes geometric and positional priors of the corresponding instrument instance.

To construct an instance-level feature that integrates both semantic and spatial cues, we project ${\overset{\sim }{b}}_{t}^{i}$, into a high-dimensional space using a fully connected layer, as shown in Equation (4):

(4) $$p_{t}^{i}=FC\left({\overset{\sim }{b}}_{t}^{i}\right),$$

and concatenate it with the semantic region feature, as shown in Equation (5):

(5) $$b_{t}^{i}=Concat\left(f_{t}^{i},\;p_{t}^{i}\right).$$

This results in a position-aware instance representation $z_{t}^{i}\in R^{d}$, which provides a compact yet interpretable summary of each instrument spatial and appearance characteristic.

### 3.4. Instance-Level Spatiotemporal Consistency Modeling

To capture temporal evolution and maintain category consistency across frames, we design a lightweight transformer encoder that processes instance features over time.

Given a sequence of T consecutive frames, the input feature set is $Z=\left\{z_{t}^{i}|t=1,T;i=1,N_{t}\right\}$. We first apply temporal position encoding PE(t) to preserve frame order, then feed the features into a multi-head self-attention encoder, as shown in Equations (6) and (7):

(6) $$h_{t}^{i}=MHSA\left(z_{t}^{i}+\;PE\left(t\right)\right),$$

(7) $${\overset{\sim }{h}}_{t}^{i}=FFN\left(h_{t}^{i}\right),$$

where MHSA denotes the multi-head self-attention operation and FFN is a feed-forward layer.

This process enables contextual aggregation across both temporal and instance dimensions, ensuring consistent representation for the same instrument in different frames.

Finally, the refined instance feature ${\overset{\sim }{h}}_{t}^{i}$ is passed through a classification head to predict the instrument category, as shown in Equation (8):

(8) $${\hat{y}}_{t}^{i}=Softmax\left(W_{c}{\overset{\sim }{h}}_{t}^{i}+b_{c}\right),$$

and the classification loss is defined as Equation (9):

(9) $$L_{cls}=-\frac{1}{N}\sum_{i=1}^{N}y_{t}^{i}log\left({\hat{y}}_{t}^{i}\right).$$

The overall loss function for joint optimization is given by Equation (10):

(10) $$L=L_{seg}+\lambda L_{cls},$$

where λ balances segmentation and classification objectives.

### 3.5. Implementation Details

All experiments were conducted on an NVIDIA RTX 4090 GPU (NVIDIA, Santa Clara, CA, USA) with PyTorch 2.1. The model was trained with an AdamW optimizer using an initial learning rate of 1 × 10^−4^, a weight decay of 1 × 10^−5^, and a batch size of 4. Each video sequence was sampled with 5 consecutive frames (T = 5) for temporal modeling. The bounding box projection dimension was set to 256, matching the region feature dimension. We trained for 80 epochs, with early stopping based on validation mIoU.

We evaluate our method on the EndoVis 2017 Robotic Instrument Segmentation and EndoVis 2018 Robotic Scene Segmentation datasets. Following standard practice, we report the metrics mIoU, IoU, and mcIoU for performance comparison.

The proposed model requires 65M parameters and 98 GFLOPs per frame sequence, comparable to MATIS (96 GFLOPs) and TraSeTr (102 GFLOPs), while maintaining faster inference (12.5 fps on RTX 4090).

## 4. Results

### 4.1. Datasets

We train and evaluate our method on two publicly available experimental frameworks, the Endovis 2017 and Endovis 2018 [25] datasets. Each dataset consists of 10 video sequences of porcine abdominal surgery. Each video contains 300 frames with a sampling frequency of 2 Hz and a resolution of 1280 × 1024. 8 × 225 frames are used as the training set, and the remaining 8 × 75 frames and 2 × 300 frames are used as the test set. For a fair comparison with previous methods, we follow the evaluation criteria established in [20]. We only use the 4-fold cross-validation proposed in For Endovis 2018, we use the additional instance annotations given in and their predefined training and validation splits. For evaluation, we adopt three common segmentation metrics from [20]: mean intersection over union (mIoU), intersection over union (IoU), and mean class intersection over union (mcIoU). Finally, we present the standard deviation error between Endovis 2017 folds.

### 4.2. Main Results

Table 1 shows the comparison results on Endovis 2017 and Endovis 2018. Our method outperforms all previous methods in all three overall segmentation indicators, and improves the three overall indicators of mIoU, IoU, and mcIoU by 3.06%, 2.99%, and 1.67% and 2.36%, 2.85%, and 6.06%, respectively.

### 4.3. Qualitative Results

**Figure 2:**
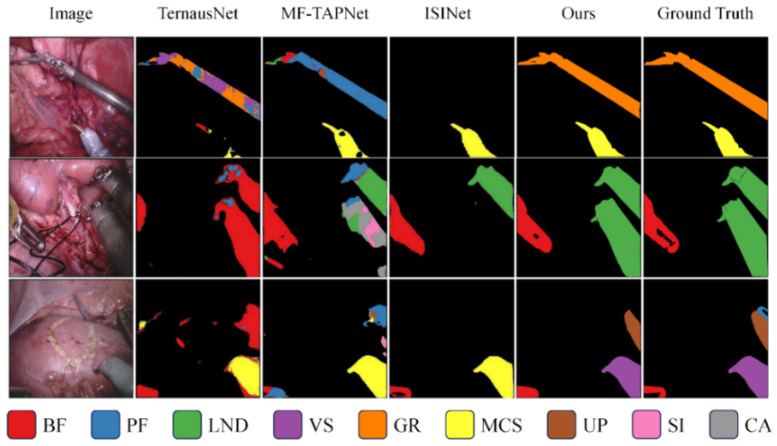
Qualitative comparison of segmentation results on the EndoVis 2017 and EndoVis 2018 datasets. Visualization of the proposed segment-then-classify framework versus pixel classification-based methods. Each color denotes a distinct surgical instrument category, and the color–instrument correspondence is shown in the legend below. Our method produces more complete and temporally coherent masks, particularly in regions with motion blur or overlapping tools.

We also visualize the segmentation results. Figure 2 shows a qualitative comparison between our method and previous pixel classification-based surgical instrument segmentation methods on the Endovis 2017 and Endovis 2018 datasets. The previous pixel-by-pixel classification strategy lacks spatial consistency, resulting in incomplete and coherent instrument masks (e.g., columns 2 and 3), or even failure to segment instruments at all (e.g., column 4). Our mask classification-based method can better utilize instance-level properties to segment complete surgical instruments.

**Figure 3:**
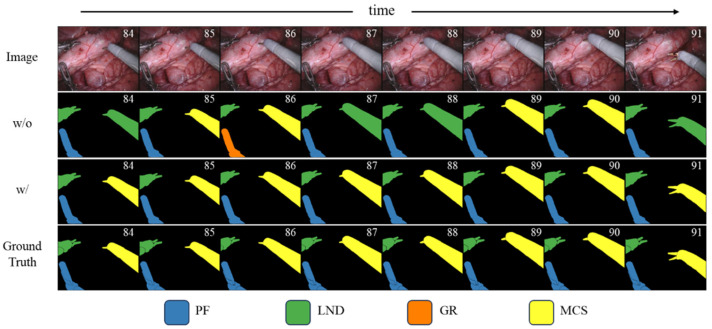
Effect of instance-level spatiotemporal consistency modeling on classification stability. Qualitative comparison of segmentation results before and after introducing instance-level spatiotemporal consistency information. Without this module, the same instrument exhibits inconsistent category predictions across consecutive frames, whereas our method maintains stable and coherent classification over time.

Figure 3 specifically shows the qualitative comparison of the results before and after the introduction of instance-level spatiotemporal consistency information. When the binary masks are the same, this paper further enhances the spatiotemporal consistency of instances between consecutive frames by introducing the instance-level spatiotemporal consistency information of the target instrument, thereby improving the classification results.

### 4.4. Ablation Experiment

We show the impact of position prior information and region proposal features on model performance when used as instance-level summary features in Table 2. Both properties improve the overall performance of the model. Among them, the position prior information has a more obvious effect on improving model performance, which also shows that interpretable position prior information plays a vital role in extracting instance motion model information.

## 5. Discussion

The experimental results on the EndoVis 2017 and 2018 datasets demonstrate the effectiveness of the proposed Segment-then-Classify framework in improving both spatial completeness and temporal stability.

Compared with pixel-wise classification methods, our approach ensures more coherent mask boundaries by decoupling segmentation from classification.

Furthermore, the incorporation of bounding box-guided priors provides explicit spatial cues that enhance interpretability and reduce temporal ambiguity, especially in cases of occlusion or fast motion.

While the method shows consistent improvements, several limitations remain. First, the current spatiotemporal modeling focuses on short-term consistency within a limited frame window, which may not fully capture long-term surgical workflow dynamics. Second, the framework assumes that instance masks are of sufficient quality; inaccurate mask generation can propagate errors into the classification stage. Third, although our model improves interpretability, it still requires considerable computational resources for transformer-based temporal encoding.

The current evaluation is limited to the EndoVis datasets, which, although standard, may not cover the full variability of clinical environments. Extending validation to other datasets such as CholecSeg8k or SurgVisDom is planned for future work to further assess generalization.

### Practical Managerial Significance

The proposed framework has potential clinical benefits in several real-world scenarios. Temporally stable and interpretable instrument segmentation can improve intraoperative awareness for robot-assisted surgery, enabling safer tool navigation and collision avoidance. It also facilitates postoperative analysis and skill assessment by providing consistent instrument tracking data. Furthermore, stable segmentation outputs can serve as reliable inputs for downstream tasks such as surgical phase recognition and workflow modeling, contributing to smarter, data-driven surgical assistance systems.

## 6. Open Research Questions and Future Directions

Despite the progress achieved, several open research questions (ORQs) remain.

(1) Long-term temporal modeling: How can transformer-based structures efficiently capture dependencies over entire surgical procedures without excessive computation?
(2) Generalization to unseen tools: Can the model adapt to novel instruments or surgical scenes without retraining, possibly through domain adaptation or few-shot learning?
(3) Multi-modal integration: How can visual segmentation be combined with kinematic or force-sensing data to improve scene understanding?
(4) Real-time deployment: What architectural or hardware-level optimizations are required to deploy the framework in real robotic surgery environments?

Addressing these questions could lead to more robust, efficient, and interpretable models for surgical video understanding.

## 7. Conclusions

In this paper, we proposed a segment-then-classify framework for surgical instrument instance segmentation in endoscopic videos. Unlike traditional pixel-wise classification methods, our approach decouples mask generation and category prediction, leading to more complete and temporally stable segmentation results. To further enhance classification accuracy, we introduced a bounding box-guided temporal modeling strategy that combines geometric priors with semantic region features. By leveraging a Transformer encoder to model instance-level spatiotemporal consistency across frames, our method effectively improves mask classification while maintaining high-quality mask structures. Extensive experiments on the EndoVis 2017 and 2018 datasets demonstrate that our framework outperforms state-of-the-art methods in both segmentation completeness and classification robustness, especially under challenging temporal conditions. In future work, we plan to extend the proposed framework toward long-term temporal modeling, multi-modal feature integration, and real-time clinical deployment in robot-assisted surgery.

## Figures and Tables

**Table 1:** Performance of the proposed method and existing approaches on the EndoVis 2017 and 2018 datasets. The best results are shown in bold. Our method achieves higher mIoU, IoU, and mcIoU scores, indicating improvements in both spatial completeness and temporal consistency.

Dataset	Method	mIoU	IoU	mcIoU	Instrument Category						
					BF	PF	LND	VS/SI	GR/CA	MCS	UP
Endovis 2017	TernausNet	35.27	12.67	10.17	13.45	12.39	20.51	5.97	1.08	1.00	16.76
	MF-TAPNet	37.25	13.49	10.77	16.39	14.11	19.01	8.11	0.31	4.09	13.40
	Dual-MF	45.80	-	26.40	34.40	21.50	**64.30**	24.10	0.8	17.90	21.80
	ISINet	55.62	52.20	28.96	38.70	38.50	50.09	27.43	2.1	28.72	12.56
	TraSeTr	60.40	-	32.56	45.20	**56.70**	55.8	**38.90**	11.40	**31.30**	18.20
	Kurmann et al. [26]	65.70	-	-	-	-	-	-	-	-	-
	MATIS	66.73	61.79	34.99	**67.17**	50.36	46.53	31.49	11.08	13.57	24.79
	Ours	**69.79**	**64.78**	**36.66**	61.03	52.28	45.69	34.66	**15.00**	20.81	**27.14**
Endovis 2018	TernausNet	46.22	39.87	14.19	44.20	4.67	0.00	0.00	0.00	50.44	0.00
	MF-TAPNet	67.87	39.14	24.68	69.23	6.10	11.68	14.00	0.91	70.24	0.57
	ISINet	73.03	70.94	40.21	73.83	48.61	30.98	37.68	0.00	**88.16**	2.16
	TraSeTr	76.20	-	47.71	76.30	**53.30**	46.50	40.60	13.90	86.20	17.15
	MATIS	82.31	77.01	48.57	83.55	38.65	40.48	92.56	70.38	0	14.39
	Ours	**84.67**	**79.86**	**54.63**	**83.95**	41.47	**66.57**	**92.74**	**74.20**	0	**23.50**

**Table 2:** Ablation of Location and Region Proposal Features for Instance-Level Summarization in EndoVis 2017. The best results are shown in bold. “✓” indicates the presence of the attribute and “-” indicates its absence.

Position Prior	Region Proposal	mIoU	IoU	mcIoU
-	-	66.73	61.79	34.99
✓	-	68.91 (+2.18)	63.86 (+2.07)	35.31 (+0.32)
-	✓	67.02 (+0.29)	62.10 (+0.31)	35.28 (+0.29)
✓	✓	**69.79** (+3.06)	**64.78** (+2.99)	**36.66** (+1.67)

## Data Availability

Restrictions apply to the availability of these data. Data were obtained from MICCAI Endoscopic Vision Challenge and are available at https://endovissub2017-roboticinstrumentsegmentation.grand-challenge.org/ (accessed on 9 September 2025) and https://endovissub2018-roboticscenesegmentation.grand-challenge.org/ (accessed on 9 September 2025) with the permission of MICCAI Endoscopic Vision Challenge.
